# Cognitive appraisal modulates Theta Burst Stimulation effects on stress-reactive rumination and affect

**DOI:** 10.3758/s13415-025-01314-z

**Published:** 2025-06-23

**Authors:** Isabell Int-Veen, Stefanie De Smet, Matias M. Pulopulos, Gert Vanhollebeke, Beatrix Barth, Sarah Pasche, Francesco Albasini, Chris Baeken, Hans-Christoph Nuerk, Christian Plewnia, Vanessa Nieratschker, Andreas Jochen Fallgatter, Ann-Christine Ehlis, David Rosenbaum, Marie-Anne Vanderhasselt

**Affiliations:** 1 Department of Psychiatry and Psychotherapy, Tübingen Center for Mental Health (TüCMH), University of Tübingen, Tübingen, Germany; 2 Department of Head and Skin, Faculty of Medicine and Health Sciences, Ghent University, Ghent, Belgium; 3 Ghent Experimental Psychiatry (GHEP) Lab, Ghent, Belgium; 4 Brain Stimulation and Cognition (BSC) Lab, Department of Cognitive Neuroscience, Faculty of Psychology & Neuroscience, Maastricht University, Maastricht, The Netherlands; 5 Department of Experimental Clinical and Health Psychology, Ghent University, Ghent, Belgium; 6 Department of Electronics and Information Systems, Ghent University, Ghent, Belgium; 7 Medical Imaging and Signal Processing (MEDISIP) Group, Ghent, Belgium; 8 German Center for Mental Health (DZPG), partner site Tübingen, Germany; 9 Central Institute of Mental Health (CIMH), Mannheim, Germany; 10 Dept. of Psychiatry and Psychotherapy, Central Institute of Mental Health, Medical Faculty Mannheim, University of Heidelberg, Heidelberg, Germany; 11 German Center for Mental Health (DZPG), partner site Mannheim-Heidelberg-Ulm, Germany; 12 Department of Psychiatry, University Hospital (UZBrussel), Brussels, Belgium; 13 Department of Electrical Engineering, Eindhoven University of Technology, Eindhoven, The Netherlands; 14 Department of Psychology, University of Tuebingen, Tuebingen, Germany

**Keywords:** Appraisal, Stress, Rumination, Trier Social Stress Test, Theta Burst Stimulation, Dorsolateral Prefrontal Cortex

## Abstract

The DLPFC plays a central role in cognitive appraisal and stress regulation, as this process—particularly secondary appraisal, where individuals assess their ability to cope with a situation—significantly influences stress responses on both psychological and physiological levels. We conducted a study where we applied different types of Theta Burst Stimulation (intermittent (i)TBS vs. continuous (c)TBS vs. sham (s)TBS) to the left DLPFC to increase or decrease the cortical excitability of the prefrontal neural network. We then examined how participants responded psychologically and physiologically to a potent psychosocial stressor, the Trier Social Stress Test (TSST), and aimed to investigate the influence of secondary cognitive appraisal on the effects of TBS on the stress response. Depending on the stimulation, we discovered that the cognitive appraisal significantly influenced rumination, positive and negative affect specifically in stress recovery. More precisely, as expected, individuals who perceived greater control experienced a faster recovery of stress-reactive ruminative thinking following sTBS and lower rumination following the stressor. We found lower increases and faster recovery of negative affect in all stimulation conditions and faster decreases in positive affect after the TSST following cTBS and iTBS, suggesting beneficial effects of both stimulation conditions. Concerning the assessed physiological variables, namely heart rate, heart rate variability, and salivary cortisol, we did not observe any impact of appraisal. These findings suggest that the effect of secondary appraisal on psychological variables depends on the type of stimulation, and while it may be linked to increased DLPFC activity, further research is needed to clarify the neurostimulation mechanisms involved.

**Supplementary Information:**

The online version contains supplementary material available at 10.3758/s13415-025-01314-z.

## Introduction

Stress is a complex phenomenon that emerges when individuals perceive a threat to their available resources (Lazarus, 1966; Lazarus & Folkman, 1984). This perception is heavily influenced by cognitive appraisal, which Lazarus and Folkman (1984) defined as “an evaluative process that determines why and to what extent a particular transaction or series of transactions between the person and the environment is stressful” (Lazarus & Folkman, 1984, p. 19). Subsequently, the differentiation of two types of cognitive appraisal, primary and secondary appraisal, has emerged. According to the definition by Velichkovsky (2009), primary appraisal "[…] is concerned with the evaluation of how (potentially) harmful a particular situation is. Secondary appraisal is concerned with the evaluation of whether the individual possesses the resources to successfully face the demands of the situation" (Velichkovsky, 2009, p. 543). Within this framework, stress is experienced when a situation is perceived as more threatening or relevant to the individual compared with the resources available to them.

Cognitive appraisal has been shown to influence the stress response on a psychological and physiological level and most probably accounts for a substantial portion of the variance in interindividual responses to stressors (Dickerson & Kemeny, 2004). The first studies examining the influence of cognitive appraisal on the stress response utilized very diverse and partly nonstandardized stressors (Maier et al., 2003; Rohrmann et al., 1999; Tomaka et al., 1993) and assessment methods of cognitive appraisal. With the Primary Appraisal Secondary Appraisal Scale (PASA) by Gaab (2009), a psychometrically validated questionnaire was developed for the use with the Trier Social Stress Test (TSST; Kirschbaum et al., 1993). The TSST is a standardized, ecologically valid and potent stressor, that has been shown to reliably induce stress on a psychological and physiological level (Allen et al., 2014; Dickerson & Kemeny, 2004). Employing the TSST, Pulopulos and colleagues (2020a) conducted a study in which participants received either positive or negative bogus feedback on their ability to cope with psychosocial stressors before completing the task. Participants who received negative feedback on their ability to deal with stress perceived the situation as more threatening, challenging, and having fewer resources to deal with the situation, and after controlling for self-efficacy, they also showed higher stress-induced cortisol responses. Higher secondary appraisal was also related to lower reductions in heart rate variability (HRV) during stress anticipation. Furthermore, in a study by Juster et al. (2012), PASA stress index (primary appraisal minus secondary appraisal) significantly predicted increases in salivary cortisol due to the TSST—but not blood pressure—and steeper recovery of cortisol and blood pressure levels (Juster et al., 2012). The authors interpret the latter finding in terms of a priming effect that causes heightened vigilance and increased physiological readiness to respond in participants with increased anticipatory stress. Another study found higher increases in total cortisol in expectation of the TSST in the case of lower control expectations (Het et al., 2009). Interestingly, in their second experiment, they could not replicate these findings but observed the threat subscale significantly predicted the reactivity of cortisol.

When considering the neural underpinnings of the aforementioned constructs, the regulation of the stress response involves several interconnected neural pathways linking the prefrontal cortex with the amygdala (Ulrich-Lai & Herman, 2009), among others. One key Region of Interest (ROI) is the Dorsolateral Prefrontal Cortex (DLPFC), which is functionally connected with the amygdala (Berboth & Morawetz, 2021), and an important region of the Central Executive Network (CEN), which is activated during higher-order cognitive functions, for instance, attention, working memory, and decision-making and comprises further regions, such as the Dorsomedial Prefrontal Cortex and the Posterior Parietal Cortex (Hermans et al., 2014; Seeley et al., 2007; Van Oort et al., 2017). Summarizing previous findings, van Oort et al. (2017) conducted a recent systematic review of 35 studies examining functional connectivity and activity in response to acute stress, proposing an inverse U-shaped relationship between stress and activation in the CEN. According to this proposed relationship, the activation of the CEN and associated regions, such as the DLPFC, is highest under moderate stress and decreases under both lower and extreme stress levels.

The crucial role of the DLPFC in stress appraisal and stress regulation is also reflected in a recently postulated theory by De Raedt and Hooley: According to the Neurocognitive framework for Regulation Expectation (De Raedt & Hooley, 2016), individuals with high expectations of being able to deal with the future situation will engage in proactive stress anticipation on a psychological, behavioral, and physiological level. This may be reflected by the use of effective emotion regulation strategies, with the increased anticipatory activity of the DLPFC and as a consequence adaptive stress regulation. The authors further highlight the importance of stress anticipation in the development of stress-related disorders, such as depression. Indeed, aberrant functioning of the DLPFC has been repeatedly observed in patients with Major Depressive Disorder (MDD) (for a review see Pizzagalli & Roberts, 2022). Most research, however, has so far focused on activation of the DLPFC during the confrontation with a stressor or in response to it. Interestingly, in studies using the TSST, the aforementioned prefrontal hypoactivation was observed not only in patients with MDD but also in healthy individuals with a tendency to ruminate (Rosenbaum et al., 2021, 2018a, 2018b). Ruminative thinking itself also impacts the stress response, because it is associated with higher heart rate, blood pressure, and cortisol responses (Ottaviani et al., 2016).

Intuitively, several studies aimed to investigate the causal impact of the DLPFC on the stress response by using Non-Invasive Brain Stimulation (NIBS). For instance, Baeken and colleagues (2014) found that a single session of high-frequency repetitive Transcranial Magnetic Stimulation (HF-rTMS) over the left DLPFC resulted in significantly lower increases in cortisol levels during the Critical Feedback Task. Also using the same task and HF-rTMS over the left DLPFC, Remue and colleagues (2016) observed significant increases in HRV. Using the TSST and HF-rTMS over the left DLPFC, Pulopulos & colleagues (2020b) could replicate the findings of Baeken et al. (2014) by observing significantly lower stress-reactive cortisol levels in the active vs. control group. In contrast, there were no differences in stress-induced changes in HRV and mood between the two groups. These studies highlight the potential role of NIBS, particularly targeting the DLPFC, in modulating the stress response and underscore the need for further investigation into its mechanisms. There is considerable variability in the effects of rTMS, which may be influenced by psychological and cognitive factors linked to the functionality and involvement of the DLPFC; however, further research is needed to investigate the underlying mechanisms.

To the best of our knowledge, this is the only study employing NIBS in the framework of anticipatory cognitive appraisal, which is why we conducted a randomized sham-controlled Theta Burst Stimulation (TBS) study. In an exploratory analysis, we investigated the impact of secondary cognitive appraisal on the effects of intermittent (iTBS), continuous (cTBS), and sham TBS (sTBS) on the psychological (subjective stress, positive and negative affect, state rumination) and physiological stress response (heart rate, HRV, salivary cortisol). Our focus is specifically on secondary appraisal, because it aligns more closely with our research interest in understanding how individuals assess their resources and coping abilities in response to stress. Moreover, the sense of control central to secondary appraisal is closely linked to the prefrontal control network and plays a key role in processes of emotion regulation.

We expected to find attenuated increases in subjective stress, state rumination, negative affect, heart rate, and salivary cortisol and attenuated decreases in positive affect and HRV following iTBS compared with sTBS, reflecting its excitatory effect on the DLPFC. In contrast, we expected increased stress responses (e.g., larger increases in subjective stress, state rumination, negative affect, heart rate, and salivary cortisol and more pronounced decreases in positive affect and HRV) following cTBS compared with sTBS owing to assumed inhibitory effects of cTBS on the left DLPFC. We further hypothesized that iTBS would lead to a faster recovery of the stress response compared with sTBS and cTBS, while recovery might be slower following cTBS. We hypothesized that participants with greater feelings of control (i.e., higher secondary appraisal) would exhibit dampened stress responses on a physiological level (dampened cortisol response and a reduced decline in anticipatory HRV), and we explored how secondary appraisal impacts the effects of TBS, stress regulation, and rumination.

## Methods

### Participants

We recruited right-handed, healthy volunteers aged 18 to 35 via postings at the university hospital and on social media. For a comprehensive list of inclusion criteria, we refer to supplementary material S1. All eligible participants received invitations to join the study. We initially aimed to recruit 138 participants based on an a priori power analysis. However, for various reasons (see supplementary material S2), we excluded 11 participants (3 in Ghent and 8 in Tübingen). This resulted in a final sample of 127 participants, of which 62.2% were female, with an average age of 22.21 years (*SD* = 2.99). Participants were randomly assigned between-subjects to their TBS condition: iTBS (*n* = 39), cTBS (*n* = 43), or sTBS (*n* = 45).

### Procedure

**Fig. 1:**
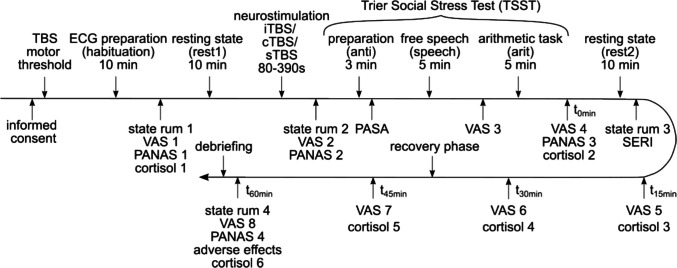
Experimental procedure. cTBS = continuous Theta Burst Stimulation; ECG = electrocardiogram; iTBS = intermittent Theta Burst Stimulation; PANAS = Positive and Negative Affect Schedule; PASA = Primary Appraisal Secondary Appraisal Scale; SERI = State Emotion Regulation Inventory; state rum = state rumination questionnaire; sTBS = sham Theta Burst Stimulation; VAS = Visual Analogue Scale assessing current stress (0–100%)

Experimental sessions occurred at either the university hospitals in Ghent or Tübingen at specific times (12:30, 13:30, 16:00, or 17:00) to account for cortisol’s circadian rhythm. Participants provided written consent before determining their resting motor threshold (RMT) and being prepared for electrocardiogram (ECG) measurement. The stimulation location was marked using the Beam F3 localization method. Following a 10 min habituation, participants underwent a 10 min resting state, followed by baseline assessments of positive and negative affect, state rumination and subjective stress, along with the first salivary sample. Depending on their stimulation condition, participants received iTBS, cTBS, or sTBS. Afterwards, stress, state rumination, and affect were assessed before the stress induction via the TSST. After reading the instructions to prepare a speech about their strengths and qualifications, participants completed the PASA before they had 3 min to prepare and take notes. Then, two experimenters wearing white coats entered the room. After delivering the speech (5 min), participants gave another stress rating and then had to complete an arithmetic task (5 min). Following this, participants’ affect, state rumination, subjective stress, and salivary cortisol levels were assessed again. An hour after the TSST, affect, state rumination, subjective stress, and participants’ beliefs regarding their stimulation condition were assessed. Finally, participants were debriefed and compensated (Fig. 1).

### Salivary cortisol

We assessed salivary cortisol levels (measured in nmol/L) at six different points during the experiment using Salivettes (Sarstedt AG & Co., REF 51.1534.500). Participants were instructed to place the swab in their mouth for 2 min. After storing the samples at -20 °C, they were thawed and centrifuged for 4 min at 1000 g. Subsequently, we conducted enzyme linked immunosorbent assays (IBL International, Cortisol ELISA, REF RE52611) in duplicate, following the manufacturer's guidelines. The coefficients of variation for both intra- and inter-assay measurements were < 10%.

### Theta Burst Stimulation

Participants underwent a single session of TBS targeting the left DLPFC, with coil positioning determined using the Beam F3 method. RMT was assessed as the minimum TMS intensity needed to elicit a motor response in the right abductor pollicis brevis in at least five of ten attempts (Rothwell et al., 1999). Stimulation intensity was set at 80% of RMT (Huang et al., 2005), with no significant differences observed between the cTBS (*M* = 39.4, *SD* = 7.45), iTBS (*M* = 39.58, *SD* = 8.93), and sTBS (*M* = 38.73, *SD* = 7.28) groups. However, participants in Ghent received significantly higher stimulation intensities (*M* = 43.95, *SD* = 7.13) compared with Tübingen (*M* = 34.56, *SD* = 5.25). Stimulation parameters included a total of 1200 pulses at a frequency of 50 Hz and burst frequency 5 Hz. During cTBS, there was an 80 s train of uninterrupted TBS including 400 bursts of 3 pulses, and the iTBS protocol consisted of 40 cycles of 2 s Theta Burst trains (10 bursts of 3 pulses each) followed by 8 s of rest (i.e., a total of 390 s; McCalley et al., 2021). Please note that the duration and end of cTBS and iTBS were aligned as a waiting period was inserted prior to the cTBS. sTBS was delivered with a coil mimicking active stimulation. Site-specific differences of TBS devices are elaborated in supplementary material S3. Participants were seated in Fowler’s position, blindfolded, wore ear protection, and were naive to their stimulation condition. To ensure double blinding, a study nurse uninvolved in the experiment applied the stimulation.

### Electrocardiogram

Heart rate was recorded by using a one-channel ECG (for details see also supplementary material S4). All ECG data underwent manual artifact checks and were analyzed with Kubios HRV Premium version 3.5.0 (Tarvainen et al., 2014). To enhance accuracy, we applied medium correction to identify incorrect beats (e.g., ectopic) and employed cubic spline interpolation to replace misidentified or missing values (Tarvainen et al., 2014). We assessed HRV by calculating the Root Mean Square of Successive Differences (RMSSD) in milliseconds. RMSSD is less susceptible to motion artifacts and respiratory influences compared with other HRV indices (Laborde et al., 2017; Penttilä et al., 2001). To maintain consistency, we calculated RMSSD for 5 min time epochs, resulting in a total of 16 epochs. Similarly, we calculated mean heart rates in 5 min epochs. Because our focus was on changes in heart rate across different phases of the experiment, such as stress reactivity and recovery, we averaged the RMSSD and mean heart rate epochs during baseline and recovery, yielding one measure per phase per participant (De Smet et al., 2021). For instance, during the baseline phase, we averaged 2 epochs of 5 min, resulting in one mean value per baseline. In the recovery phase, each set of three (or two for the last phase) consecutive 5 min epochs was averaged into one mean value, resulting in four recovery phase values: 0–15 min post TSST, 15–30 min post TSST, 30–45 min post TSST, and 45–55 min post TSST.

### Trier Social Stress Test

The TSST (Kirschbaum et al., 1993) was used to induce psychosocial stress. Participants were instructed to imagine they had applied for a job, with part of the interview requiring them to prepare and deliver a speech about their personal strengths and qualifications. After a brief preparation period (3 min), they delivered the speech (5 min) to two jury members (experimenters) dressed in white lab coats, who remained unresponsive to any attempts at social interaction. Following the speech, participants faced an unexpected arithmetic challenge, counting aloud backward in steps of 13 from 2081 for 5 min. To heighten social evaluative pressure, they were instructed to maintain eye contact with a jury member and perform as quickly and accurately as possible. Errors required restarting from the initial number, and participants were consistently urged to improve their speed and precision, regardless of their performance. To further increase stress, participants were informed that their performance was being video-recorded for later evaluation by behavioral experts. They were not told the overall duration of the speech and arithmetic task, enhancing their sense of unpredictability and lack of control.

### Cognitive appraisal

We assessed participants' cognitive appraisal using the Primary Appraisal Secondary Appraisal Scale (PASA; Gaab, 2009; Gaab et al., 2005), a self-report questionnaire specifically designed to evaluate cognitive appraisal in the anticipation of stress. Participants provided ratings for 16 self-report items on a 6-point Likert-style scale, which ranged from "1 = completely disagree" to "6 = completely agree". The PASA consists of four primary scales: threat, challenge, self-concept of own abilities, and control expectancy—each comprising four items. From these, two secondary scales are derived: Primary appraisal is calculated by averaging the "threat" and "challenge" scales, while secondary appraisal is obtained by averaging the "self-concept of own abilities" and "control expectancy" scales. The overall PASA stress index is determined by subtracting the secondary appraisal score from the primary appraisal score (Stress Index = Primary Appraisal – Secondary Appraisal). The psychometric properties of the PASA scales have been shown to be satisfactory to good, with Cronbach's α ranging from 0.6–0.8 (Gaab et al., 2005).

### Subjective stress

Participants were regularly prompted to evaluate their current subjective stress using Visual Analogue Scales (VAS). These scales featured a 100-mm straight line, with one end indicating 0% ("not stressed at all") and the other end showing 100% ("very stressed"), marked at 10% intervals.

### Positive and negative affect

Participants were asked to assess their current mood by using the Positive and Negative Affect Schedule (PANAS; Watson et al., 1988). This involved rating their feelings for both positive and negative emotions using a 5-point Likert scale, where "1 = very slight" and "5 = extremely". To calculate total scores, we summed the values of all items within each subscale. In this scoring system, higher scores indicated more pronounced levels of either negative or positive affect. The PANAS demonstrates reliable results and strong internal consistency (Krohne et al., 2016). Negative affect subscale had a Cronbach's $\alpha$ of 0.86 and the positive affect subscale had a Cronbach's $\alpha$ = 0.84.

### State rumination

To assess state rumination during the session, a questionnaire by Rosenbaum and colleagues (2021, 2018a, 2018b) was utilized, demonstrating strong internal consistency (Cronbach's $\alpha$ in the current study = 0.93). Participants were asked to assess their agreement with 18 statements related to thoughts that might have arisen during the recent rest period. Responses were recorded on a 5-point Likert scale, ranging from "1 = not at all" to "5 = very often". This questionnaire incorporated items from the Perseverative Thinking Questionnaire (Ehring et al., 2011), the Ruminative Response Scale (Nolen-Hoeksema, 1991), the Amsterdam Resting State Questionnaire (Diaz et al., 2013), and a questionnaire developed by de Jong-Meyer and colleagues (2009). The items can be found in supplementary material S5. To calculate total scores, the responses were summed, with items 7 and 13 being reverse scored.

### Data analysis

We conducted data analysis in RStudio Version 2023.06.2 + 561 (RStudio Team, 2023) and R Version 4.3.1 (R Core Team, 2023). To ensure data quality, we screened for multivariate outliers by calculating Mahalanobis distances for each dependent variable (subjective stress, state rumination, positive and negative affect, heart rate, HRV, and salivary cortisol). Each distance was compared with the critical value of the chi-squared distribution at a significance level of *α* = 0.001. Cases exceeding this threshold were identified as outliers and subsequently excluded from further analysis (*n* = 0 for subjective stress, *n* = 2 for state rumination, *n* = 0 for positive, *n* = 3 for negative affect, *n* = 0 for heart rate, *n* = 6 for HRV, and *n* = 6 for salivary cortisol).

To determine the most suitable model for each variable, we fitted various models with different distributions (normal, gamma, inverse-Gaussian) using the R-packages lme4 (Bates et al., 2015) and lmerTest (Kuznetsova et al., 2017) and selected the model with the lowest Akaike Information Criterion. Specifically, we fitted a linear mixed model for subjective stress, a general linear mixed model (glm) with inverse-Gaussian distribution and log link function in the case of state rumination, heart rate and salivary cortisol, a glm with gamma-distribution and identity link function in the case of positive affect and a glm with gamma-distribution and log link function for negative affect and HRV.

Our models each included a three-way interaction of time (categorical, representing repeated assessments during the experimental session), stimulation condition (categorical, including iTBS, cTBS, and sTBS), and PASA secondary appraisal (numerical, grand mean centered). Each model also incorporated random intercepts for individual participants.

We further investigated the impact of expectancy effects (correct or incorrect identification of active or sham stimulation) by incorporating this variable as a main effect. A four-way interaction could not be estimated due to highly unequal group sizes, which rendered any interpretation unreliable.

To investigate potential effects arising from data being collected at two different locations (Ghent and Tübingen), we re-ran all analyses, including the factor of university, and report the results in supplementary material S6. The findings indicated that the inclusion of this factor did not lead to any significant changes, as the predictor was never significant and the model did not explain a significantly greater amount of variance when the factor was included.

Additionally, as an exploratory analysis we examined sex differences across all variables of interest and report these findings in supplementary material S7, as they are not the main focus of this article. Please note that effects (three-way interactions of time, condition, and PASA secondary appraisal) seemed to be primarily driven by women and were mostly not apparent in men. Specifically, this was the case for subjective stress ratings, state rumination, and positive affect. For physiological variables, we observed similar results among both sexes, namely no cognitive appraisal-dependent effects of the stimulation. We would like to note that sex-specific analyses were not initially planned, and therefore, owing to the relatively small subgroups, we urge caution in the interpretation of the results.

We reported *p*-values using type III Wald chi-squared statistics. Lower-order effects are not reported. Post-hoc tests were conducted using the R-package emmeans (Lenth & others, 2022), and we corrected for multiple comparisons using the Tukey-method. Treatment contrasts were set for categorical factors (contr.treatment; sTBS as reference category) and polynomial contrasts for numerical, ordered predictors (contr.poly).

For data visualization, we utilized the ggplot2 (Wickham, 2009) and sjPlot-package (Lüdecke, 2024). In case the three-way interaction yielded significance, we fitted separate models for each stimulation condition. To simplify the analysis and interpretation of the post-hoc tests and make them more intuitive, we further split the numerical predictor PASA into two groups based on a median split when it showed a significant interaction effect in the overall analysis.

For analysis regarding the impact of TBS on physiological and psychological outcomes of the stress response, we refer to our previous article on this data (De Smet et al., 2024).

## Results

### Descriptive statistics

First, we investigated cognitive appraisal dependent on the stimulation group (cTBS vs. iTBS vs. sTBS) and found no significant differences regarding primary appraisal, *F*(2, 124) = 0.444, *p* = 0.642, secondary appraisal, *F*(2, 124) = 1.608, *p* = 0.204, nor the PASA stress index, *F*(2, 124) = 1.159, *p* = 0.317 (Table 1). We further found no significantly different distributions of people with low vs. high primary or secondary appraisal among the stimulation groups (Table 2).

**Table 1:** Test statistics comparing participants’ PASA scores across TBS conditions

	cTBS (*n* = 43)	iTBS (*n* = 39)	sTBS (*n* = 45)	Test statistic
PASA primary appraisal (PA)	3.82 (0.92)	3.97 (0.89)	3.78 (1.03)	*F*(2, 124) = 0.444, *p* = 0.642
PASA secondary appraisal (SA)	4.20 (0.77)	3.93 (0.68)	4.17 (0.79)	*F*(2, 124) = 1.608, *p* = 0.204
PASA stress index	-1.53 (6.05)	0.17 (4.96)	-1.54 (6.29)	*F*(2, 124) = 1.159, *p* = 0.317

**Table 2:** Absolute and relative frequencies of participants with low and high PASA primary appraisal (threat and challenge) and PASA secondary appraisal (control and self-concept) dependent on the stimulation condition

		cTBS (*n* = 43)	iTBS (*n* = 39)	sTBS (*n* = 45)	Test statistic
PASA primary appraisal (PA)	Low primary appraisal (PA < 4)	23 (18.11%)	20 (15.75%)	24 (18.89%)	${\upchi }^{2}$(2) = 0.049, *p* = 0.976
	High primary appraisal (PA ≥ 4)	20 (15.75%)	19 (14.96%)	21 (16.54%)	
PASA secondary appraisal (SA)	Low secondary appraisal (SA < 4)	16 (12.6%)	17 (13.39%)	16 (12.6%)	${\upchi }^{2}$(2) = 2.46, *p* = 0.292
	High secondary appraisal (SA ≥ 4)	27 (21.26%)	22 (17.32%)	29 (22.83%)	

The relative number refers to the percentage of the total sample (*N* = 127)

### Blinding and expectation effects

A binomial test revealed that participants guessed the correct stimulation condition above chance level (64% correct, *p* = 0.002), with differences between study sites. Blinding was successful in Ghent (56%, *p* = 0.374) but not in Tübingen (72%, *p* < 0.001). In Tübingen, participants in the cTBS condition correctly identified active stimulation above chance (*p* < 0.001), but sTBS was not distinguishable from active stimulation at either site (*p* > 0.05).

Running the analysis of each of our dependent variables by including the (in)correct guesses of the stimulation condition as a main effect did not reveal significant influence on the study results (all *p-*values > 0.145).

### Subjective stress

**Fig. 2:**
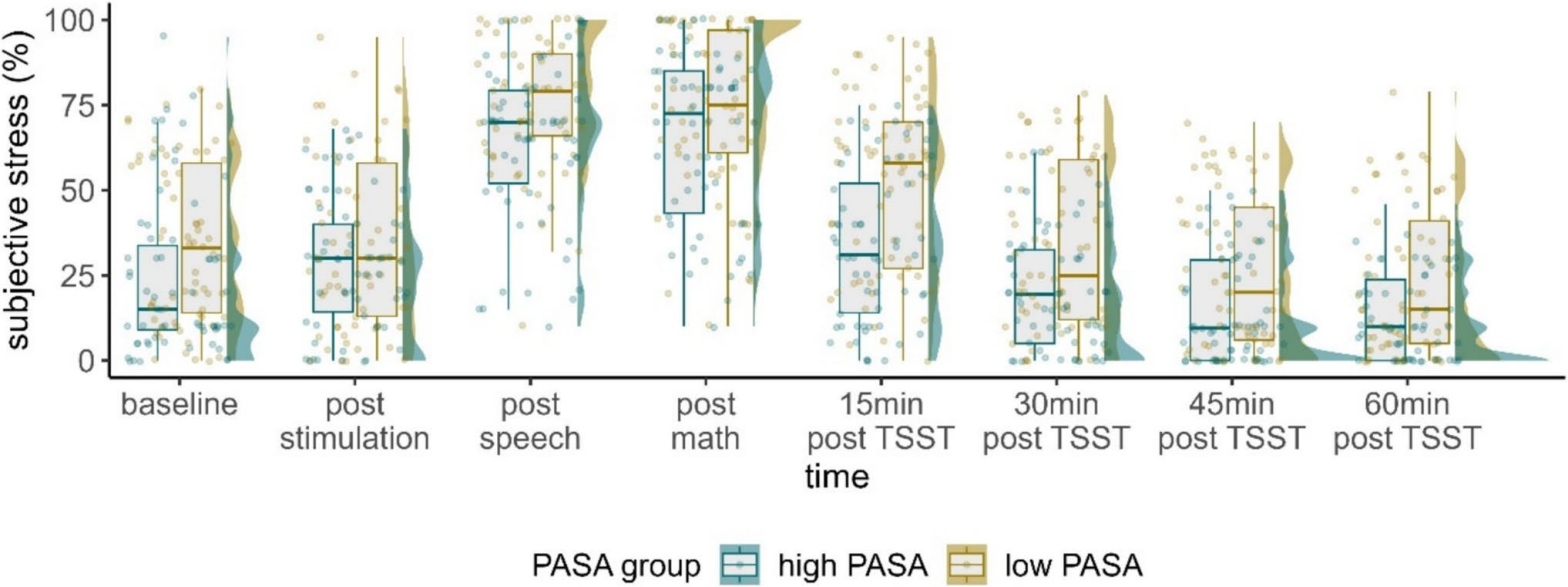
Raincloud plots for subjective stress dependent on PASA secondary appraisal. Note that in terms of clearer visualization, we categorized continuous PASA secondary appraisal in low and high scores according to a median split. Individual jittered raw data are represented by dots. Data distributions are depicted by split-half violin plots (on the right side of each series of boxplots). Each boxplot displays the stimulation condition median alongside the interquartile ranges (horizontal lines). min = minutes; post speech = post job interview of the TSST; post math = post arithmetic task of the TSST; TSST = Trier Social Stress Test

We observed an interaction of PASA secondary appraisal with time, ${\upchi }^{2}$(7) = 16.943, *p* < 0*.*05, φ = 0.37. Post-hoc tests, in which the numerical PASA secondary appraisal predictor was split into two groups based on the median, revealed a significant difference between the groups. Higher PASA secondary appraisal (indicating higher resources) was associated with lower subjective stress at all time points (all *t*-values < -2.17, all *p*-values < 0.03, all *d's* < -0.57), except immediately after the stimulation (*t*(323) = − 1.721, *p* < 0.086, *d* = -0.45), where both groups reported comparable stress levels (Fig. 2).

We further observed a significant main effect of condition, ${\upchi }^{2}$(2) = 6.612, *p* < 0.05, φ = 0.42. After correction for multiple comparisons using the Tukey-method, this effect was reflected by marginally significantly lower subjective stress following sTBS compared with cTBS (contrast sTBS-cTBS: *t*(121) = -2.106, *p* = 0.093, *d* = -0.48).

### State rumination

A significant three-way-interaction of time, stimulation condition, and PASA secondary appraisal was found, ${\upchi }^{2}$(6) = 16.935, *p* < 0.01, φ = 0.37. Fitting separate models for each stimulation condition, the interaction of time and PASA secondary appraisal only yielded significance in the sTBS model, ${\upchi }^{2}$(3) = 19.039, *p* < 0.001, φ = 0.65. Post-hoc tests with the PASA secondary appraisal group predictor yielded significantly lower state rumination 60 min post TSST in the group of participants with higher PASA secondary appraisal compared with the group of participants with lower PASA secondary appraisal (*z* = -2.182, *p* < 0.05, *d* = -9.25).

**Fig. 3:**
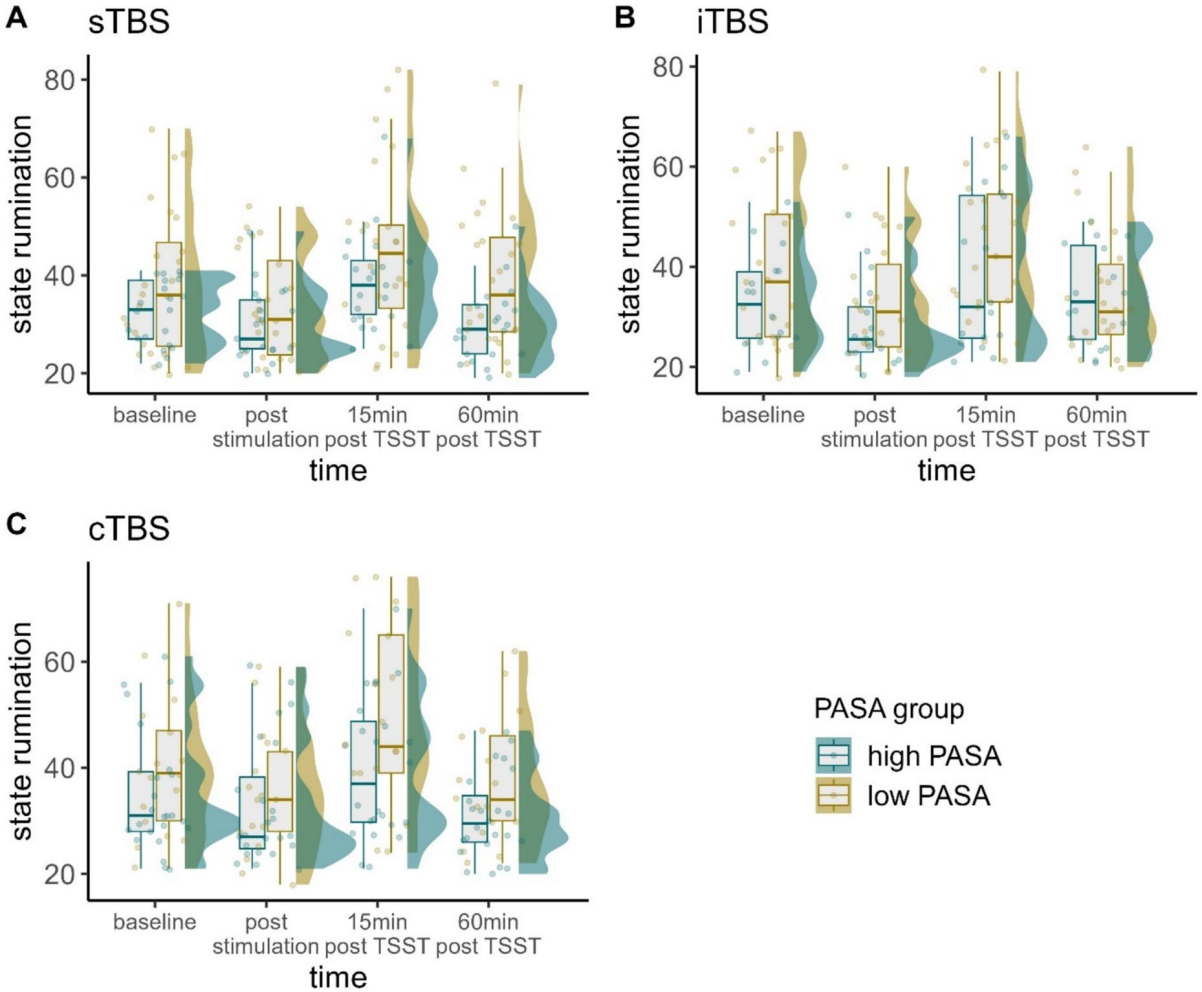
Raincloud plots for state rumination dependent on stimulation condition (A = sTBS, B = iTBS, C = cTBS) and PASA secondary appraisal. Note that in terms of clearer visualization, we categorized continuous PASA secondary appraisal in low and high scores according to a median split. Individual jittered raw data are represented by dots. Data distributions are depicted by split-half violin plots (on the right side of each series of boxplots). Each boxplot displays the stimulation condition median alongside the interquartile ranges (horizontal lines). cTBS = continuous Theta Burst Stimulation; iTBS = intermittent Theta Burst Stimulation; min = minutes; sTBS = sham Theta Burst Stimulation; TSST = Trier Social Stress Test

**Fig. 4:**
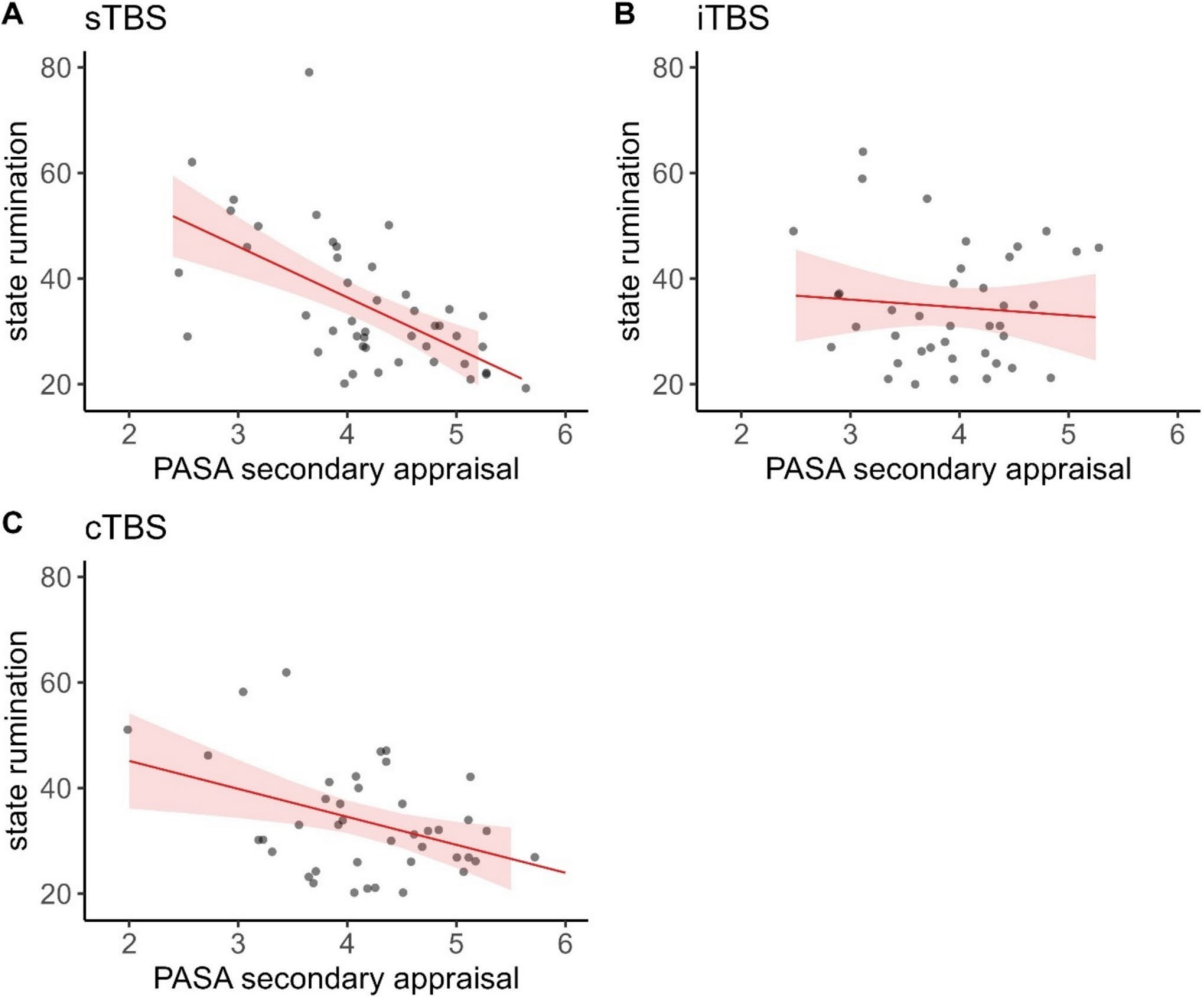
Predicted values (marginal effects) for state rumination post 60 min TSST dependent on PASA secondary appraisal ratings for each stimulation condition (**A** = sTBS, **B** = iTBS, **C** = cTBS), respectively. cTBS = continuous Theta Burst Stimulation; iTBS = intermittent Theta Burst Stimulation; sTBS = sham Theta Burst Stimulation; PASA = Primary Appraisal Secondary Appraisal Scale

Both groups exhibited significant increases in state rumination from baseline to 15 min after the TSST, as well as from post-TBS to 15 min post TSST (all *z*-values < -3.759, all *p*-values < 0.01, all *d*'s < -8.153) and significant decreases again at 60 min post TSST (all *z*-values < -3.759, all *p*-values < 0.01, all *d*'s < -5.592). Only in participants with high secondary appraisal (feelings of control), we observed a significant decrease in state rumination between baseline and 60 min post TSST (*z* = 2.586, *p* < *0.*05, *d* = 3.52). Lastly, only in participants with low secondary appraisal, we additionally observed significant decreases between baseline and post-stimulation (*z* = 2.822, *p* < 0.05, *d* = 3.72) and increases also between post-stimulation and 60 min post TSST (*z* = -3.693, *p* < 0.01, *d* = -4.96). In the iTBS and cTBS model, only time yielded significance (Figs. 3 and 4).

### Positive affect

There was a significant three-way-interaction of time, stimulation condition, and PASA secondary appraisal, ${\upchi }^{2}$(6) = 21.070, *p* < 0.01, φ = 0.41. Fitting separate models for each stimulation condition, the interaction of time and PASA secondary appraisal yielded significance in case of participants having received cTBS, ${\upchi }^{2}$(3) = 37.690, *p* < 0.001, φ = 0.94, and iTBS, ${\upchi }^{2}$(3) = 10.795,* p* < 0.05, φ = 0.53.

Post-hoc tests of the iTBS model with the PASA group predictor yielded only marginally significant differences between low and high secondary appraisal at 60 min after the TSST. Namely, participants with lower feelings of control experienced higher positive affect (*z* = -1.692, *p* = 0.091, *d* = -21.04). Regarding the changes between the time points, we observed overall decreases in positive affect between baseline and 60 min post TSST in both groups (all *z*-values > 2.644, all *p*-values < 0.05, all *d*'s> 17.4). Participants with higher secondary appraisal experienced significant decreases also between post-stimulation and 60 min post TSST (*z* = 3.802, *p* < 0.001, *d* = 26.85) and between 0 min post TSST and 60 min post TSST (*z* = 4.569, *p* < 0.001, *d* = 33.98). Participants with lower secondary appraisal experienced significant decreases also between baseline and 0 min post TSST (*z* = 3.854, *p* < 0.001, *d* = 24.70) and between post-stimulation and 0 min post TSST (*z* = 3.116, *p* < 0.01, *d* = 19.69).

Post-hoc tests of the cTBS model indicated significantly lower positive affect in case of lower resources directly after the TSST (*z* = 2.785, *p* < 0.01, *d* = 32.21). Interestingly, the time courses appeared to be reversed dependent on PASA scores. Participants with higher feelings of control experienced increases in positive affect between baseline and the end of the TSST (*z* = -3.276, *p* < 0.01, *d* = -21.55) and post-stimulation and the end of the TSST (*z* = -4.181, *p* < 0.001, *d* = -26.71) and decreases again between the end of the TSST and 60 min post TSST (*z* = 4.559, *p* < 0.001, *d* = 28.82). Participants with lower feelings of control conversely experienced decreases in positive affect between baseline and the end of the TSST (*z* = 2.986, *p* < 0.05, *d* = 20.43) and post-stimulation and the end of the TSST (*z* = 3.156, *p* < 0.01, *d* = 22.01). They also experienced decreases between baseline and 60 min post TSST (*z* = 3.419, *p* < 0.01, *d* = 22.80) as well as between post stimulation and 60 min post TSST (*z* = 3.598, *p* < 0.01, *d* = 24.38).

**Fig. 5:**
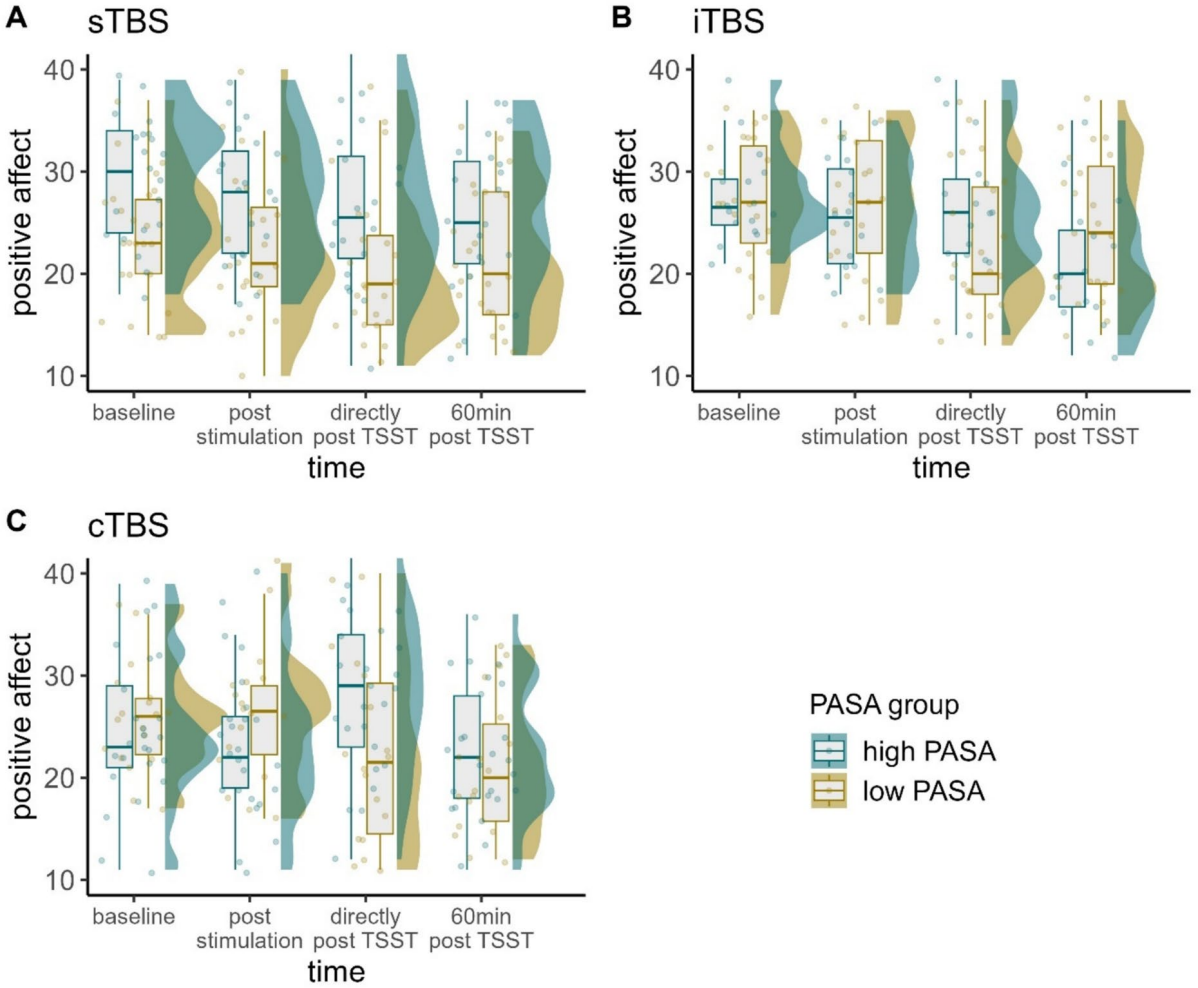
Raincloud plots for positive affect dependent on stimulation condition (A = sTBS, B = iTBS, C = cTBS) and PASA secondary appraisal. Note that in terms of clearer visualization, we categorized continuous PASA secondary appraisal in low and high scores according to a median split. Individual jittered raw data are represented by dots. Data distributions are depicted by split-half violin plots (on the right side of each series of boxplots). Each boxplot displays the stimulation condition median alongside the interquartile ranges (horizontal lines). cTBS = continuous Theta Burst Stimulation; iTBS = intermittent Theta Burst Stimulation; min = minutes; sTBS = sham Theta Burst Stimulation; TSST = Trier Social Stress Test

**Fig. 6:**
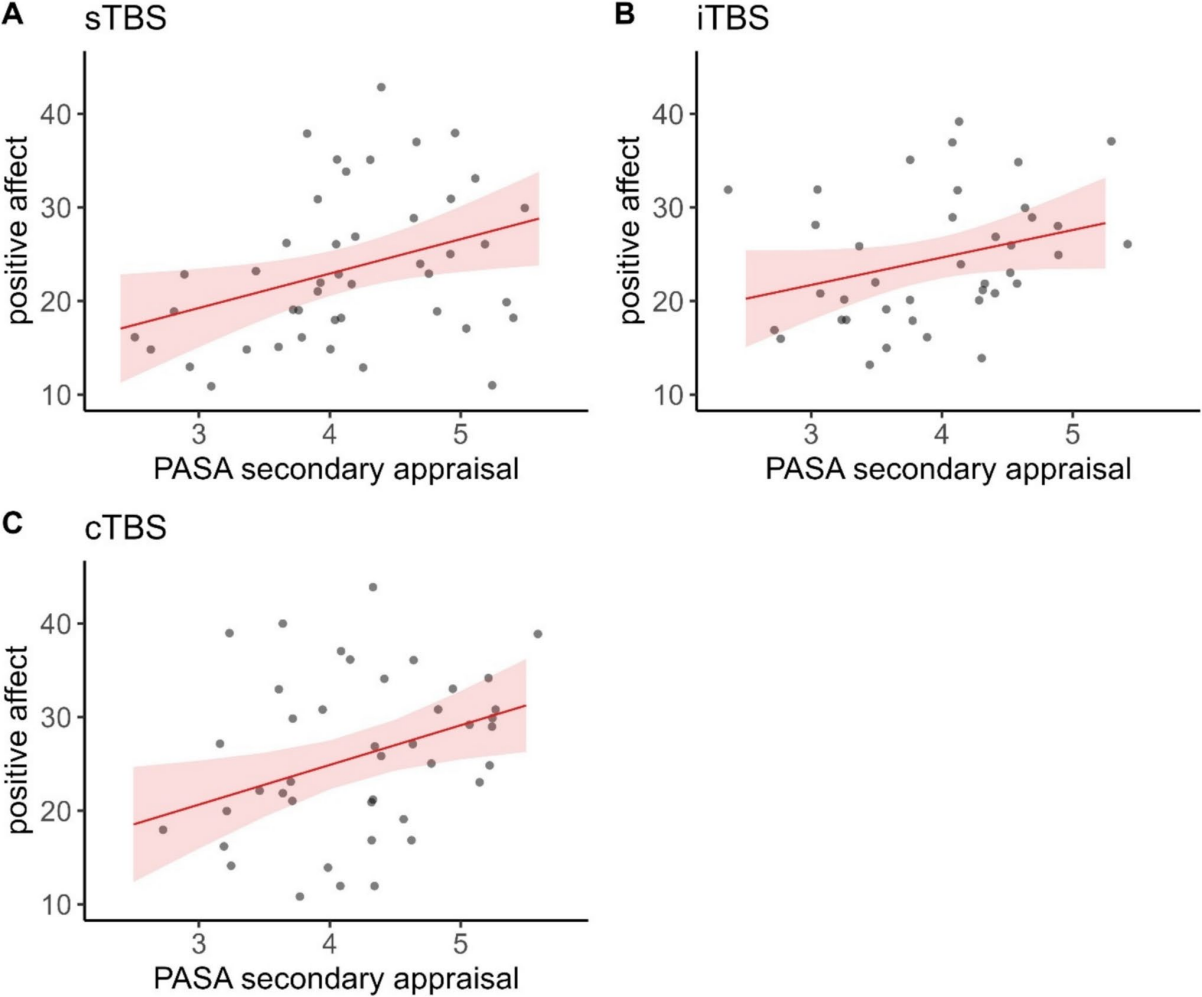
Predicted values (marginal effects) for positive affect 0 min post TSST dependent on PASA secondary appraisal ratings for each stimulation condition (A = sTBS, B = iTBS, C = cTBS), respectively. cTBS = continuous Theta Burst Stimulation; iTBS = intermittent Theta Burst Stimulation; sTBS = sham Theta Burst Stimulation; PASA = Primary Appraisal Secondary Appraisal Scale

In the sTBS model only time yielded significance (Figs. 5 and 6).

### Negative affect

Fitting our model we observed a significant three-way interaction of time, stimulation condition, and PASA secondary appraisal, ${\upchi }^{2}$(6) = 22.022, *p* < 0.01, φ = 0.42. Fitting separate models for each stimulation condition, the interaction of time and PASA secondary appraisal yielded significance in each model (sTBS: ${\upchi }^{2}$(3) = 12.405, *p* < 0.01, φ = 0.53; iTBS: ${\upchi }^{2}$(3) = 10.721, *p* < 0.05, φ = 0.52; cTBS: ${\upchi }^{2}$(3) = 8.752, *p* < 0.05, φ = 0.46).

Post-hoc tests with the PASA group predictor indicated that participants with lower secondary appraisal experienced higher negative affect at baseline following cTBS (*z* = -2.365, *p* < 0.05, *d* = -1.25), as well as directly after the TSST following iTBS (*z* = -2.216, *p* < 0.05, *d* = -1.29) and cTBS (*z* = -2.351, *p* < 0.05, *d* = -1.24) and 60 min post TSST following sTBS (*z* = -2.748, *p* < 0.01, *d* = -1.27) and cTBS (*z* = -2.165, *p* < 0.05, *d* = -1.14).

**Fig. 7:**
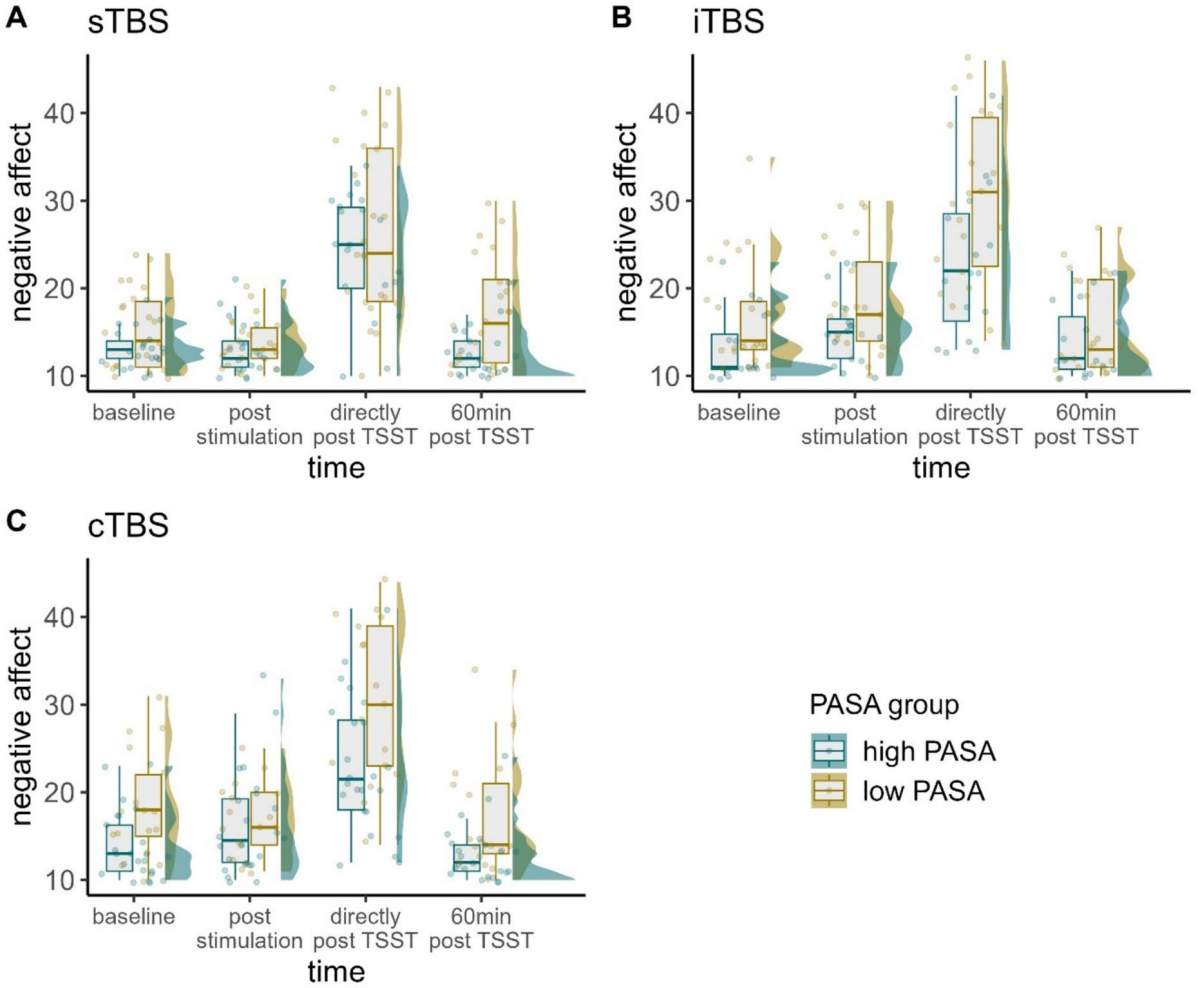
Raincloud plots for negative affect dependent on stimulation condition (A = sTBS, B = iTBS, C = cTBS) and PASA secondary appraisal. Note that in terms of clearer visualization, we categorized continuous PASA secondary appraisal in low and high scores according to a median split. Individual jittered raw data are represented by dots. Data distributions are depicted by split-half violin plots (on the right side of each series of boxplots). Each boxplot displays the stimulation condition median alongside the interquartile ranges (horizontal lines). cTBS = continuous Theta Burst Stimulation; iTBS = intermittent Theta Burst Stimulation; min = minutes; sTBS = sham Theta Burst Stimulation; TSST = Trier Social Stress Test

**Fig. 8:**
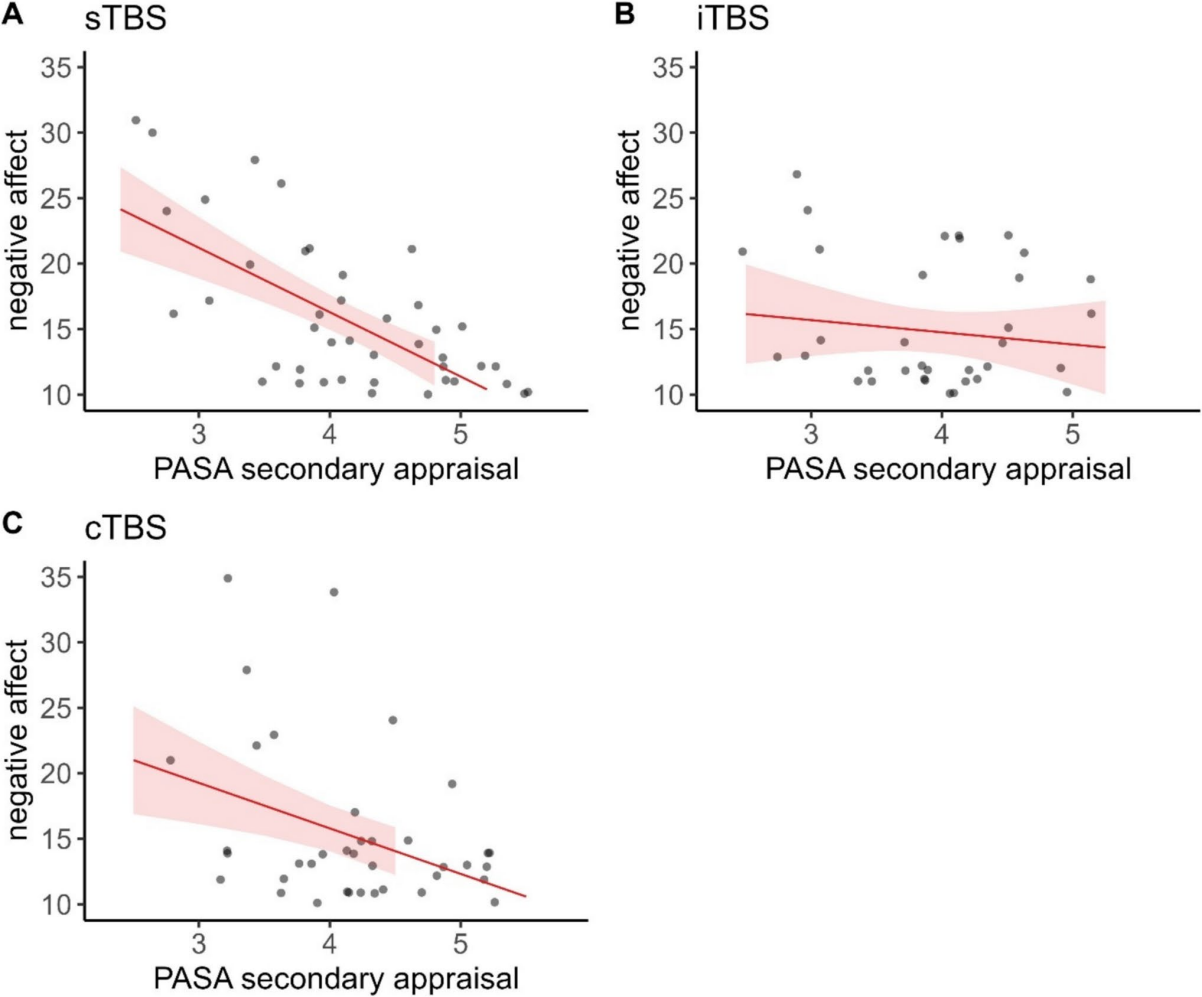
Predicted values (marginal effects) for negative affect 60 min post TSST dependent on PASA secondary appraisal ratings for each stimulation condition (A = sTBS, B = iTBS, C = cTBS). cTBS = continuous Theta Burst Stimulation; iTBS = intermittent Theta Burst Stimulation; sTBS = sham Theta Burst Stimulation; PASA = Primary Appraisal Secondary Appraisal Scale

Regarding the differences between time points, we observed very similar results in the different TBS models and PASA groups. In all three models and both groups, we observed significant increases in negative affect between baseline and 0 min post TSST (all *z*-values < − 7.667, all *p*-values < 0.001, all *d*'s < -2.28), as well as between post-stimulation and 0 min post TSST (all *z*-values < -5.866, all *p*-values < 0.001, all *d*'s < -1.75) and significant decreases again between 0 min post TSST and 60 min post TSST (all *z*-values > 7.325, all *p*-values < 0.001, all *d*'s > 1.98). Only in the sTBS and iTBS models, we observed significant increases between post-stimulation and 60 min post TSST in case participants experienced lower secondary appraisal (all *z*-values < -2.984, all *p*-values < 0.015, all *d*'s < -0.8). Only in the cTBS model, we additionally observed significant increases between baseline and post-stimulation (*z* = -2.628, *p* < 0.05, *d* = -0.66) and decreases between post-stimulation and 60 min post TSST in the group experiencing higher feelings of control (*z* = 3.696, *p* < 0.05, *d* = 0.93) (Figs. 7 and 8).

### Heart rate

Fitting the model, we only observed a significant main effect of time, ${\upchi }^{2}$(7) = 1141.333, *p* < 0.001, φ = 3.08, indicating a significant increase due to the TSST and decrease again post-stress.

### Heart rate variability

As with heart rates, we only observed a significant main effect of time, ${\upchi }^{2}$(7) = 398.388, *p* < 0.001, φ = 1.87, indicating a significant decrease due to the TSST and increase again post-stress.

### Salivary cortisol

We observed a significant interaction of time and stimulation condition, ${\upchi }^{2}$(10) = 18.999,* p* < 0.05, φ = 0.4, but no effect of PASA secondary appraisal.

## Discussion

Stress is a multifaceted phenomenon that arises when individuals perceive a threat to their resources (Lazarus, 1966; Lazarus & Folkman, 1984). Secondary cognitive appraisal determines whether individuals believe to have the resources to cope with challenging situations (e.g., threat). So far, results on the impact of appraisal on the psychological and physiological stress response remain scarce and inconclusive. With its inhibitory connections to the amygdala, the DLPFC is a key ROI in the investigation of the stress response and stress regulation. According to the Neurocognitive Framework for Regulation Expectation (De Raedt & Hooley, 2016), individuals with high expectancies of being able to deal with stressful situations (i.e., higher secondary appraisal) will engage in proactive stress anticipation on a psychological, behavioral and physiological level, increased anticipatory activity of the DLPFC, and as a consequence adaptive stress regulation. The impact of anticipatory appraisal on the effect of TBS on the stress response warrants further exploration. In our study, we applied a single session of randomized, placebo-controlled TBS (between-subjects iTBS vs. cTBS vs. sTBS) on the left DLPFC in a total of 127 healthy individuals.

Our results indicate an appraisal-dependent impact of the stimulation (i.e., significant three-way interactions of time, stimulation condition, and PASA secondary appraisal) on all psychological variables assessed except for subjective stress. Owing to the scarcity of studies investigating the influence of neurostimulation and cognitive appraisal on state rumination and affect, we were unable to formulate specific hypotheses and instead conducted exploratory analyses, which align with intuitive expectations. For stress-reactive state rumination, we observed a faster recovery following the stress induction and significantly lower state rumination 60 min post TSST in participants with higher PASA secondary appraisal (i.e., higher perceived control over the situation) but only if participants had received sTBS. In the sTBS condition, only participants with higher feelings of control showed overall decreases in state rumination and less pronounced increases due to the TSST, while those with lower feelings of control did not. This aligns well with appraisal theory as well as our initial expectations, which suggest that higher secondary appraisal can have protective effects on stress responses.

We did not find any time-dependent effects of secondary appraisal when participants received an active stimulation, which was not in line with our expectation. Active TBS might function as a "perturbation" of the stress-recovery in terms of ruminative thinking, making the recovery process less reliant on individual cognitive factors such as perceived control. It remains inconclusive why cTBS and iTBS did not affect the psychological variables differently due to their putatively contrary neural effects. A recent meta-analysis by Kirkovski and colleagues (2023) examined the neurobiological effects of TBS on neural activity and functional connectivity during resting-state measurements and tasks requiring executive functions, i.e., cognitive control. They demonstrated that stimulation of the prefrontal cortex with cTBS and iTBS can each evoke both excitatory and inhibitory responses. The authors attribute this variability in the neurobiological effects of TBS to differences in localization methods, leading to varying levels of targeting accuracy, as well as to the complexity of the neural organization of the frontal cortex and the demands associated with the performed tasks. Many influencing factors are likely still unknown today, while others, such as genetic factors or age, have already been identified (Corp et al., 2020; Ridding & Ziemann, 2010). Unfortunately, this study did not include neural data to confirm the anticipated impact on the left DLPFC, limiting the interpretation of the absent or inconsistent stimulation-dependent effects of cognitive appraisal.

Interestingly, in comparison to state rumination, where the effect is evident only in the sTBS condition (and absent in cTBS and iTBS), we observed significant interactions of secondary appraisal and time for positive affect in the cTBS and iTBS conditions, and for negative affect in all three TBS conditions. Again, primarily stress recovery seemed to be influenced.

For positive affect, the time courses were similar for participants with both lower and higher feelings of control. In the iTBS condition, we observed only a marginally significant increase in positive affect 60 min after the TSST for participants with lower feelings of control compared with no increases for participants with higher feelings of control. This effect may potentially reflect a sense of relief within this group. Furthermore, participants with higher secondary appraisal (feelings of control) experienced significant decreases in positive affect in the recovery period of the TSST, whereas participants with lower secondary appraisal did not. Following cTBS, the time courses appeared to be reversed dependent on PASA scores (secondary appraisal). Participants with higher feelings of control experienced increases in positive affect between baseline and the end of the TSST and decreases again between the end of the TSST and 60 min post TSST, whereas participants with lower feelings of control conversely experienced decreases throughout the whole experimental procedure. These opposite effects of the TBS were generally in line with our expectation.

For negative affect, we observed an appraisal-dependent impact of the stimulation in the case of all three stimulation conditions. Higher secondary appraisal (i.e., higher perceived control) was associated with lower increases in negative affect due to the stress induction and a faster recovery post-stress. Generally, time courses of negative affect seemed to be very similar among the stimulation conditions and secondary appraisal groups. That means, higher perceived control had a beneficial effect independent of the stimulation.

In a supplementary analysis, the above-mentioned effects appeared to be primarily driven by women, suggesting a stronger effect in this subgroup. However, because sex-specific analyses were not pre-planned and the subgroup sizes are small, these findings should be interpreted with caution.

Subjective stress was the only psychological variable where no appraisal-dependent impact of the stimulation was observed, but only an interaction of time and secondary appraisal as well as a main effect of stimulation condition. Higher secondary appraisal was associated with lower subjective stress at all time points, except immediately after the stimulation. The significant main effect of condition was reflected by marginally significantly lower subjective stress following sTBS compared with cTBS. Please note that the latter effect was only marginally significant after correction for multiple comparisons and therefore should only be interpreted with caution. Moreover, because the difference is not specific to the post-stimulation time point, it does not indicate a direct effect of TBS but general group differences. Had this effect occurred exclusively after active TBS, one possible explanation could have been the stronger sensory stimulation associated with active TBS compared with sham.

Potentially, some of the differences in the reported results may arise due to differences in the assessments. For example, positive and negative affect were measured using an established questionnaire (Positive and Negative Affect Schedule), while stress-reactive rumination was assessed with a self-developed questionnaire. It is conceivable that different aspects were taken into account during the different ratings by the participants; particularly internal control beliefs, as well as variables that potentially influence them, could have had a different or varying degree of influence on the variables. The results regarding the stimulation conditions and the appraisal-dependent mechanism of left DLPFC stimulation do not appear easy to capture. Nevertheless, our data indicate an appraisal-dependent impact of the stimulation on the psychological stress response and the need for further research.

Concerning the assessed physiological variables, namely heart rate, HRV, and salivary cortisol levels, we did not observe any (appraisal-dependent) effects of the stimulation. More specifically, we only observed overall time-dependent alterations, indicating a successful stress induction. This is well in line with very recent findings of Pulopulos et al. (2020a, b) where the authors induced different expectancy effects concerning participants’ ability to be able to cope with the following TSST using explicit feedback. The authors did not find significant differences in HRV or salivary cortisol for the different feedback groups (high vs. low expectancy to cope well with the stressor). Interestingly, after controlling for general self-efficacy, they observed significant group differences concerning induced expectations for salivary cortisol. This highlights the importance of other psychological variables in the realm of anticipation of stressors and expectancy effects. Further studies are needed to disentangle the aforementioned associations. For instance, it might be possible that individual differences, such as personality traits or coping styles, interacted with the perception of control (i.e., secondary appraisal) even though we did not observe significant differences in secondary appraisal dependent on the stimulation condition. Nevertheless, some individuals may be more responsive to beliefs of control, while others may not be as affected. This idea is currently speculative, as no existing studies provide evidence to support it, underscoring the need for future research. Please note that we only recruited participants naive to NIBS in order to control for the potential impact of previously received stimulation on expectancy effects and our dependent variables; however, future studies should further elaborate other variables, e.g., self-efficacy, with respect to their potential impact on the aforementioned associations.

As already mentioned, one limitation of the current study is the absence of neural data to directly assess the effects of the TBS on brain activity. However, we are addressing this limitation by conducting a follow-up study with a similar setup (Int-Veen, Eßer, et al., n.d.; Int-Veen, Täglich, et al., n.d.), utilizing functional Near-Infrared Spectroscopy (fNIRS) to investigate the impact of the different TBS protocols (iTBS vs. cTBS vs. sTBS) on cortical oxygenation. Preliminary results of these studies indicate that the stimulation primarily exerts a consistent effect on functional connectivity (FC), particularly within regions of the CEN (Int-Veen, Täglich, et al., n.d.). At the neural level, iTBS enhances the synchrony of frontoparietal brain regions, whereas the effects of cTBS appear more variable, which is also generally in line with the findings of Kirkovski et al. (2023).

Corresponding changes in FC were also observed by Gratton et al. (2013) who aimed to investigate the neural changes following TBS applied to different target regions of mutually independent brain networks associated with cognitive control (however, please find an exemplary discussion on inconsistent naming of brain networks associated with executive function in Witt et al., 2021). Using a within-subjects design the authors applied inhibitory cTBS to these areas, targeting the cingulo-opercular network (left anterior insula), the frontoparietal network (left DLPFC), and the primary somatosensory cortex and observed increases in resting-state FC within regions of the frontal, parietal, and cingulate cortex following frontal stimulation but not following stimulation of the left primary somatosensory cortex. Their findings suggest that acute disruption of cognitive control regions by TBS leads to widespread changes in network connectivity beyond the targeted regions.

Moving away from the context of acute stress and further supporting a general mechanism regarding emotion regulation, a recent fMRI study on threat processing has shown that the (right) DLPFC plays a crucial role in the top-down regulation of emotion (Patel et al., 2024). Together with findings that variations in connectivity within the CEN are associated with cortisol levels (Peters et al., 2019) and unique connectivity patterns are also linked to the subjective appraisal of affect (Lindquist et al., 2016; Rohr et al., 2013), it seems reasonable to assume complex mechanisms between changes in FC as well as respective implications on a cognitive and affective level; however, further studies are needed to disentangle these findings.

Another point to consider when interpreting the aforementioned results is that hormonal contraceptives reduce cortisol responses relative to naturally cycling females (Gervasio et al., 2022). In this study, all recruited females were taking oral contraceptives. As a result, the variance in stress-reactive salivary cortisol levels might be reduced and potential (stimulation-dependent) effects of cognitive appraisal are diminished. Interestingly, Gaab and colleagues (2005) explicitly investigated the informative value of primary and secondary cognitive appraisal in contrast to general personality factors and found an additional value of their PASA in explaining stress-reactive cortisol increases. It remains unclear whether for instance current depressed mood might mask expectancy effects. General convictions and beliefs but most probably also the current state of mind is potentially influencing the stress response. This is also a current debate in the research field of NIBS (Schutter et al., 2023). Future research should explore participants' subjective beliefs, cognitive appraisals of the stimulation, and perceptions of stress to gain a more holistic understanding. It is important to note that we re-ran our models for each dependent variable, incorporating expectancy effects as a main effect but did not observe a significant effect. Specifically, we included whether participants were correct or incorrect in their judgment at the end of the experiment regarding whether they received real or sham stimulation. We did not explicitly manipulate expectancy effects, which resulted in relatively small subgroups when considering the number of participants in each stimulation condition, preventing more complex analyses. It is likely that the effects do not fundamentally shift the baseline but are rather time-dependent. For more complex analyses, larger groups and potentially experimentally manipulated expectancy effects are desperately needed.

## Conclusions

Our findings highlight a stimulation-dependent influence of cognitive appraisal on psychological outcomes during stress recovery. Greater perceived control over the stressful situation was associated with faster recovery of stress-reactive rumination following sTBS and reduced rumination levels overall. Negative affect showed smaller increases and faster recovery across all stimulation conditions dependent on greater perceived control, while cTBS and iTBS were linked to more rapid decreases in positive affect after the TSST. Importantly, appraisal did not affect physiological measures such as heart rate, HRV, or salivary cortisol. These results suggest that secondary appraisal predominantly impacts psychological variables, with the role of neurostimulation requiring further investigation.

## Supplementary Information

Below is the link to the electronic supplementary material.

Supplementary file1 (PDF 162 KB)

## Data Availability

The data and material are avaiable from the first and last authors upon reasonable request.

## Code availability

The code is avaiable from the first and last authors upon reasonable request.

## Declarations

### Open Practices Statements

The data and materials for the experiment reported here is available; none of the experiments was preregistered.
