# Supplementary material for: Cognitive appraisal modulates Theta Burst Stimulation effects on stress-reactive rumination and affect
Source: Cogn Affect Behav Neurosci. 2025 Jun 23;25(5):1400–18. doi: 10.3758/s13415-025-01314-z (PMC12464076; doi:10.3758/s13415-025-01314-z)
Supplement: Supplementary file 1 — Supplementary file1 (PDF 162 KB) [file 13415_2025_1314_MOESM1_ESM.pdf]

Supplementary material

Supplementary Material S1: Inclusion criteria

Inclusion criteria included: 1) age between 18 and 35 years old, 2) right-handed, 3) no history or current psychiatric or neurological disorders, 4) no cardiovascular diseases, 5) no drugs or medication use that affects mood, cognitive functioning or cardiovascular activity including antidepressant drugs, benzodiazepines and Z-drugs, 6) smoking no more than 10 cigarettes a day, 7) normal or corrected to normal vision, 8) no history of serious head injuries, brain surgery or epileptic seizures, 9) no metal implants or fragments in the head, 10) no cochlear implants or pacemaker, 11) no pregnancy, 12) no contraindications for theta burst stimulation (TBS, see also Rossi et al., 2009, 2011) and 13) naive to any form of transcranial magnetic stimulation (TMS). Moreover, given the influence of sex hormones on heart rate variability (e.g., Schmalenberger et al., 2020), only females taking hormonal contraceptives were included.

Supplementary Material S2: Details on dropouts

We had to exclude eleven participants from the final sample, with three from Ghent and eight from Tübingen. In Ghent, one participant was excluded because of being already familiar with the Trier Social Stress Test (TSST), and two female participants were excluded because they were not currently using hormonal contraceptives during the experimental session. In Tübingen, three participants missed their appointment for the experimental session, two were excluded due to technical problems during the session, two participants dropped out due to experiencing discomfort during the stimulation, and one participant was excluded because the stress induction became too overwhelming.

Supplementary Material S3: Differences in TBS-devices between the study sites

In Ghent, Theta Burst Stimulation (TBS) was administered using a figure-eight shaped coil (Magstim 70 mm double air film coil) connected to a Magstim Rapid² Plus¹ magnetic stimulator (Magstim Company Limited, Wales, UK). The sham TBS in Ghent utilized a placebo version of the Magstim figure-eight coil designed to replicate the somatosensory sensations associated with active TBS but without causing any lasting effects.

In Tübingen, a MagVenture MagPro X100 Stimulator (MagVenture, Farum, Denmark) was employed, along with a MagVenture Cool-B65 Active/Placebo coil. This figure-eight shaped coil was specifically designed for double-blind stimulation. It automatically switched between active and sham modes by flipping the coil according to device instructions. In addition, two pre-gelled surface electrodes (28x20mm) were placed around the stimulation area, providing a mild current for a superficial sensation during both the sham TBS and active TBS. For the sham TBS in Ghent, a fixed sham duration equivalent to the duration of the iTBS (i.e., 390 seconds) was utilized. In Tübingen, the sham duration matched either the duration of the cTBS (i.e., 80 seconds) or iTBS (i.e., 390 seconds), and this allocation was balanced among participants.

Crucially, participants were unable to identify sham from real stimulation as correct guesses in the sham condition were not significantly different from chance, for both study sites (57% correct $sTBS_{Ghent}$, p=0.664 and 54% correct $sTBS_{Tübingen}$, p=0.839) and different sham durations (55% correct $sTBS_{390s}$, p=0.728 and 58% correct $sTBS_{80s}$, p=0.774).

Supplementary Material S4: Differences in ECG-assessments

In Tübingen, three Ag/AgCl ring electrodes (8 mm diameter) were placed on the participants: one above the right collarbone, one below the left costal arch, and one on the neck for reference. We used a BrainAmp MR plus amplifier and Brain Vision recorder software (Brain Products, Munich, Germany) at a 1000 Hz sampling rate to record the ECG-signal.

In Ghent, two Ag/AgCl electrodes (42x24mm) were positioned beneath the left and right clavicle, with one ground electrode under the ribs for reference. The ECG signal was recorded using a Biopac MP160 system with an ECG100D smart amplifier, sampled at 1000Hz, and monitored with Biopac Acqknowledge software 5.5 (Biopac Systems Inc., USA)**.**

Supplementary Material S5: Items of the state rumination questionnaire

Table 1. Items of the state rumination questionnaire

Item	Statement
1	I repeatedly thought about my problems.
2	I kept thinking about things that bother me.
3	I dwelled on my thoughts without coming to a solution.
4	I got lost in my negative thoughts.
5	I had difficulties holding on to my thoughts.
6	I could not let go of my negative thoughts.
7	I was present.
8	I thought about why I acted wrong in certain situations.
9	I thought why I have problems other people don´t have.
10	I thought about whereby I deserved my current life situation.
11	I thought why I can´t handle things better.
12	I thought about all my shortcomings, failings, faults, mistakes.
13	I could switch between my thoughts flexibly.
14	I thought about past situations that I regret.
15	I blamed myself.
16	I got lost in thoughts about the past.
17	I was consumed by my problems and worries.
18	I couldn´t let go of my negative thoughts.

Supplementary Material S6: Investigation of the impact of university

- **Subjective stress:** Adding university as an additional main effect to the model did did not affect the significance of the other predictors and did not yield a significant additional predictor, $\chi^2(1)=3.187$, $p=0.074$, nor did the more complex model explain significantly more variance according to a Likelihood-Ratio-Test, $\chi^2(1)=3.330$, $p=0.068$.
- **State rumination**: Adding university as an additional main effect to the model did did not affect the significance of the other predictors and did not yield a significant additional predictor, $\chi^2(1)=1.040$, $p=0.308$, nor did the more complex model explain significantly more variance according to a Likelihood-Ratio-Test, $\chi^2(1)=1.020$, $p=0.313$.
- **Positive affect**: Adding university as an additional main effect to the model did did not affect the significance of the other predictors and did not yield a significant additional predictor, $\chi^2(1)=1.653$, $p=0.199$, nor did the more complex model explain significantly more variance according to a Likelihood-Ratio-Test, $\chi^2(1)=1.587$, $p=0.208$.
- **Negative affect**: Adding university as an additional main effect to the model did did not affect the significance of the other predictors and did not yield a significant additional predictor, $\chi^2(1)=2.876$, $p=0.090$, nor did the more complex model explain significantly more variance according to a Likelihood-Ratio-Test, $\chi^2(1)=2.815$, $p=0.094$.
- **Heart rate**: Adding university as an additional main effect to the model did did not affect the significance of the other predictors and did not yield a significant additional predictor, $\chi^2(1)=0.540$, $p=0.462$, nor did the more complex model explain significantly more variance according to a Likelihood-Ratio-Test, $\chi^2(1)=0.537$, $p=0.464$.
- **Heart rate variability**: Adding university as an additional main effect to the model did did not affect the significance of the other predictors and did not yield a significant additional predictor, $\chi^2(1)=0.000$, $p=0.998$, nor did the more complex model explain significantly more variance according to a Likelihood-Ratio-Test, $\chi^2(1)=0.000$, $p=0.999$.
- **Salivary cortisol**: Adding university as an additional main effect to the model did did not affect the significance of the other predictors and did not yield a significant additional predictor, $\chi^2(1)=0.581$, $p=0.446$, nor did the more complex model explain significantly more variance according to a Likelihood-Ratio-Test, $\chi^2(1)=0.581$, $p=0.446$.

Supplementary Material S7: Investigation of sex differences

First, we examined the assumptions for the analysis by testing whether the number of women and men was equally distributed across the different experimental conditions, and whether there were differences in the PASA scores (see tables 2 and 3). Subsequently, we fitted all models separately for women and for men. Please note that, for the sake of clarity, we will only report post-hoc tests comparing different PASA groups and TBS conditions at the respective time points, excluding comparisons between time points (e.g., changes from baseline to the TSST).

Table 2. Contingency table of number of female and male participants per TBS condition

	**cTBS** (*n*=43)	**iTBS** (*n*=39)	**sTBS** (*n*=45)	**Statistics**
female	27	26	26	$\chi^2(1)=0.581$, $p=0.446$
male	16	13	19	

Table 3. Contingency table of number of female and male participants per with low and high PASA primary appraisal and PASA secondary appraisal scores

		**female** (*n*=79)	**male** (*n*=48)	**Statistics**
PASA primary appraisal (PA)	low primary appraisal (PA<4)	38	29	$\chi^2(1)=1.356$, $p=0.244$
	high primary appraisal (PA≥4)	41	19	
PASA secondary appraisal (SA)	low secondary appraisal (SA<4)	45	33	$\chi^2(1)=0.715$, $p=0.398$
	high secondary appraisal (SA≥4)	34	15	

Subjective stress. When we fitted the model separately for men, we observed only a significant main effect of time, $\chi^2(7)=276.805$, $p<0.001$, and no other signifianct effects. For women, we observed a significant three-way interaction of time, PASA secondary appraisal and condition, $\chi^2(14)=30.422$, $p<0.01$. We then fitted the same model dependent on stimulation condition and observed a significant interaction of time and PASA secondary appraisal in case females received sTBS, $\chi^2(7)=19.288$, $p<0.01$, as well as cTBS, $\chi^2(7)=22.681$, $p<0.01$.

In case females received sTBS, differences between low and high PASA secondary appraisal emerged following the stress induction: 15min post TSST up until 60min post TSST, females with higher PASA secondary appraisal exhibited lower subjective stress (all *p*'s<0.05).

In case females received cTBS, differences between low and high PASA secondary appraisal emerged directly after the TBS: Again, up until 30min post TSST, females with higher PASA secondary appraisal exhibited lower subjective stress (all *p*'s<0.05).

State rumination. When we fitted the model separately for men, we observed only a significant interaction of time and condition, $\chi^2(6)=13.829$, $p<0.05$. For women, we observed a significant three-way interaction of time, PASA secondary appraisal and condition,

$\chi^2(6)=24.222$, $p<0.001$. We then fitted the same model dependent on stimulation condition and observed a significant interaction of time and PASA secondary appraisal in case females received sTBS, $\chi^2(3)=17.168$, $p<0.001$, as well as cTBS, $\chi^2(3)=17.887$, $p<0.001$.
Contrasts indicated only marginal significant differences in the sTBS condition at 60min post TSST ($p=0.056$) but significantly higher state rumination in the cTBS condition at 15 min post TSST in case of lower PASA secondary appraisal, $z=-2.374$, $p<0.05$, $d=-12.95$.

Positive affect. When we fitted the model separately for men, we observed only a significant main effect of time, $\chi^2(3)=12.115$, $p<0.001$, and no other signifianct effects. For women, we observed a significant three-way interaction of time, PASA secondary appraisal and condition, $\chi^2(6)=21.204$, $p<0.01$. We then fitted the same model dependent on stimulation condition and observed a significant interaction of time and PASA secondary appraisal in case females received iTBS, $\chi^2(3)=11.425$, $p<0.01$, as well as cTBS, $\chi^2(3)=32.456$, $p<0.001$. Contrasts indicated no significant differences between the low and high PASA secondary appraisal groups at any given time point for the iTBS condition (all *p*'s>0.166) but significantly higher positive affect directly after the TSST following cTBS for females in the high PASA secondary appraisal group compared to females in the low PASA secondary appraisal group, $z=3.552$, $p<0.001$, $d=53.88$.

Negative affect. When we fitted the model separately for men, we observed a significant three-way interaction of time, condition and PASA secondary apppraisal, $\chi^2(6)=17.016$, $p<0.01$. Post-hoc tests indicated a significant interaction of time and PASA secondary appraisal only in the iTBS condition, $\chi^2(3)=8.133$, $p<0.05$.
When we fitted the model separately for women, we also observed a significant three-way interaction of time, condition and PASA secondary apppraisal, $\chi^2(6)=14.874$, $p<0.05$. Contrasts indicated significantly higher negative affect in men in the iTBS condition in case of lower PASA secondary appraisal at baseline (i.e. previous to the TBS), $z=-1.973$, $p<.05$, $d=-1.723$, as well as directly after the TSST, $z=-2.401$, $p<.05$, $d=-2.097$.
Post-hoc tests indicated a significant interaction of time and PASA secondary appraisal only in the sTBS condition, $\chi^2(3)=14.335$, $p<0.01$. Contrasts indicated significantly higher negative affect in women in the sTBS condition 60min post TSST in case of lower PASA secondary appraisal, $z=-2.977$, $p<.01$, $d=-1.809$.

Heart rate. When we fitted the model separately for women and men, in both cases, we only observed significant main effects of time (women: $\chi^2(7)=918.403$, $p<0.001$; men: $\chi^2(7)=569.076$, $p<0.001$) but no further significant effects (all p's>0.098).

Heart rate variability. When we fitted the model separately for women and men, in both cases, we only observed significant main effects of time (women: $\chi^2(7)=323.313$, $p<0.001$; men: $\chi^2(7)=182.076$, $p<0.001$) but no further significant effects (all p's>0.305).

Salivary cortisol. When we fitted the model separately for women and men, in both cases, we only observed significant main effects of time (women: $\chi^2(5)=26.028$, $p<0.001$; men: $\chi^2(5)=29.345$, $p<0.001$). In the case of men, we further observed a significant interaction effect of time and condition, $\chi^2(10)=27.877$, $p<0.01$.

References of the Supplementary Material:

Schmalenberger KM, Eisenlohr-Moul TA, Jarczok MN, Eckstein M, Schneider E, Brenner, IG, et al. Menstrual Cycle Changes in Vagally-Mediated Heart Rate Variability are Associated with Progesterone: Evidence from Two Within-Person Studies. J Clin Med 2020;9.3:617. https://doi.org/10.3390/jcm9030617
